# The impact of physical environments on outpatient mental health recovery: A design-oriented qualitative study of patient perspectives

**DOI:** 10.1371/journal.pone.0283962

**Published:** 2023-04-19

**Authors:** Tiffany Y. Sui, Shannon McDermott, Brooke Harris, Honor Hsin

**Affiliations:** 1 Department of Adult Psychiatry, Kaiser Permanente San Jose, San Jose, CA, United States of America; 2 Department of Graduate Medical Education, Kaiser Permanente Santa Rosa, Santa Rosa, CA, United States of America; 3 Department of Graduate Medical Education, Kaiser Permanente San Jose, San Jose, CA, United States of America; Universidad Central de Chile, CHILE

## Abstract

The physical environment has been shown to affect the emotional states of patients receiving mental health treatment, yet it remains unknown whether physical space design may play a role in optimizing the delivery of mental health care. Principles of architectural design and human-centered co-design have been applied to enhance the patient experience of facility environments; however, little is known about how patients view the impact of physical spaces on their recovery. In this qualitative study, we aimed to understand patient perspectives of how physical environments contribute to mental wellbeing and personal experiences of recovery, in the context of informing future design efforts. Semi-structured telephone interviews were conducted with 13 participants receiving outpatient mental health treatment at the Kaiser Permanente San Jose Adult Psychiatry Clinic. Interviews were transcribed and themes were extracted that could inform future design concepts. The sample was comprised of nine female and three male participants, and one unidentified-gender participant, between the ages of 26–64, and across several self-reported racial/ethnic subgroups. We found four dimensions of physical environments that participants reported as impactful: 1) **sensory** design elements (colors, sounds, and textures), 2) **engagement** qualities (intensity of distracted activity such as crafting or commuting), 3) social **relational** aspects (privacy or connection), and 4) **affective experiences** evoked by being present in the space itself (feeling safe, calm, in control, self-aware, or creative was beneficial). Many of these elements were similarly noted across clinic and non-clinic environments. This study identifies key dimensions of physical environments that can serve as potential metrics of design success in supporting and facilitating mental health recovery. In the midst of the current COVID-19 pandemic, where mental health treatment has increasingly shifted outside of traditional clinics, our findings can support patients and clinicians seeking to harness potential *in situ* therapeutic benefits of physical environments.

## Introduction

Design approaches such as human-centered design (HCD) are increasingly utilized to improve patient experience in the healthcare system. HCD is an effective method for developing and refining product solutions to fit unmet human needs [1]. In one striking case study, an HCD approach toward building design of Florida Hospital for Children resulted in a profound jump in patient/family satisfaction scores from the bottom 10% of the nation, to the top 10% [2]. In mental health, there have been proposals for how architectural teams can begin conceptualizing HCD approaches in the design of the built clinic environment [3]. Previous studies have focused on acute or inpatient care settings. One qualitative study conducted with forensic patients found that patients attribute meaning to the physical space in which they receive care, and several themes identified include striving towards normalcy, feeling protected, feeling at-home, and feeling in communion with others and purpose [4]. A recent non-qualitative HCD study of user perspectives on outpatient mental health building design in Australia uncovered desired themes of belonging, self-agency, self-regulation, and comprehension of expected behaviors and spatial cues [5]. While HCD seeks to empathize with patient needs intuitively, qualitative research methods that systematically collect patient experiences and inductively identify themes of environmental influences on health recovery may provide a useful analytic counterpoint to inform future design efforts. Currently, there are no existing qualitative studies investigating patient perspectives on physical space design in outpatient mental health care globally.

The space in which patients receive mental health treatment has been found to influence how patients perceive their emotional wellbeing [3]. A recent systematic review examined the different mechanisms by which cultural ecosystem services (CESs), a term encompassing the entirety of human-environment interactions, are known to influence personal well-being, and identified 16 potential pathways of impact from the concrete (such as renumeration potential of environments) to the abstract (such as transcendent experiences of environmental immersion) [6]. Recent studies have also shown positive associations between mental health and greenspace, or urban landscapes with vegetation features [7–10]. Greenspace exposure is thought to improve mental health through “direct mediators” such as relaxing visual stimulation and “indirect mediators” such as opportunities for positive physical and social activities [8]. Most of the literature regarding greenspace has relied on cross-sectional surveying methods, however, and were focused primarily on perceived wellbeing rather than recovery from mental illnesses.

Here, we sought to conduct a rigorous, in-depth exploration of patient perspectives of physical environments, both within and external to the outpatient clinic setting, on their mental health recovery. We sought to identify themes that can meaningfully augment HCD or other applied design approaches, and we aimed to describe the different ways that physical environments can impact the mental health recovery of patients receiving care from an urban psychiatric clinic.

## Methods

This study was conducted at Kaiser Permanente San Jose in Northern California, a large community health center serving a broad population encompassing diverse socioeconomic and racial/ethnic groups, within the department of Adult Psychiatry. A few months prior to study conception, the health center had shared with the staff and research study team that a new building had been purchased for clinic space. Clinic leaders supported a design-oriented effort to investigate patient perspectives on physical environments in their mental health recovery. This study was reviewed and approved by the Kaiser Permanente Northern California Institutional Review Board (IRB).

### Participants

We used a purposeful sampling approach to gather broad and comprehensive responses. Purposeful sampling allowed us to select study participants that were accessible by phone, available to fully engage in the study, and able to articulate their experiences [11]. This approach enabled us to achieve saturation in a timely manner without sacrificing the richness of qualitative data collected.

We identified 12,320 patients that attended a mental health appointment in-person at Kaiser Permanente San Jose Adult Psychiatry over the previous 12 months of the study (virtual appointments during the COVID pandemic were acceptable, but the patient must have physically attended at least one clinic appointment on site during the 12-month period). We selected a random sample of 300 potentially eligible subjects who had received mental health care in the clinic. Additional inclusion criteria were ages 18–85 years old; having at least one clinical diagnosis of a mental health disorder; ability to read, speak, and write conversational English; and active on Kaiser Permanente’s online patient portal (kp.org). The latter two inclusion criteria reflected our recruitment process, which began with a written invitation in English (the only language spoken by study team members) sent via the online patient portal (see Procedure below). Exclusion criteria were employment by Kaiser Permanente or its affiliates; active suicidal or homicidal ideation (past ideation was acceptable); concurrent treatment in intensive outpatient program or other more intensive level of care (past treatment was acceptable); active conservatorship or guardianship by another individual or state; or receiving direct clinical care services from any member of the clinical study team.

**Fig 1:**
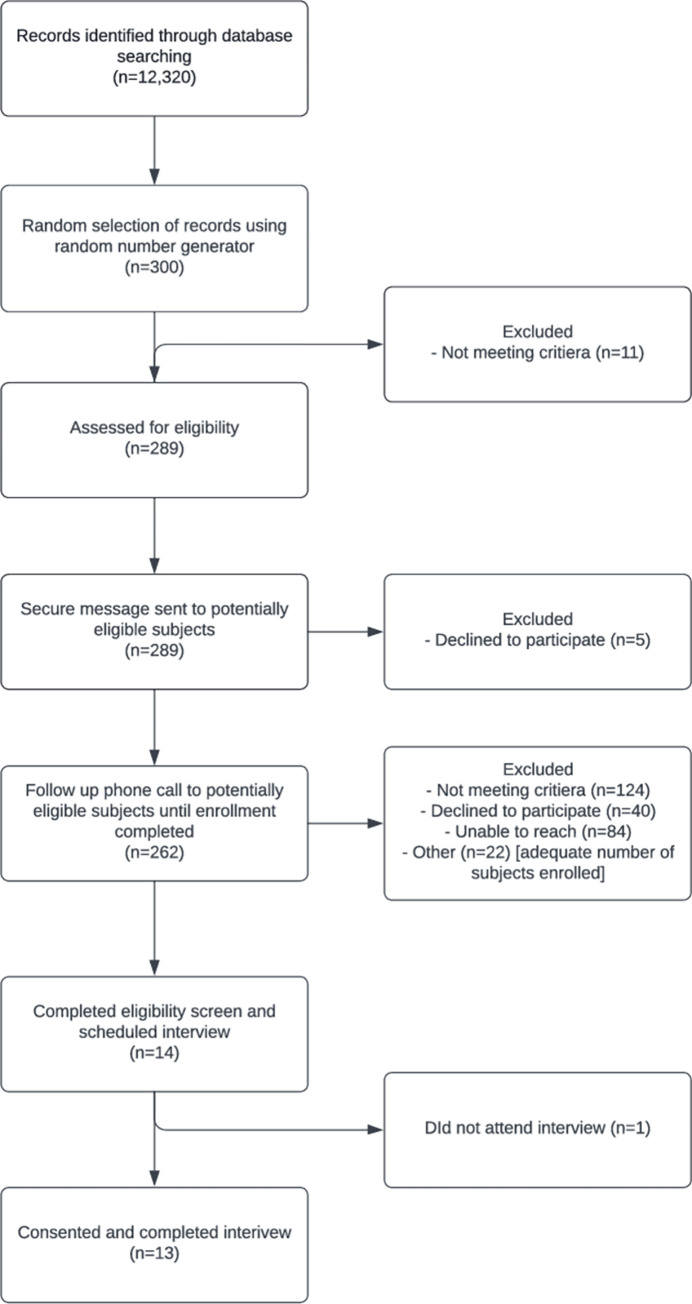
CONSORT diagram: Eligibility screening and enrollment.

Eleven of the 300 potentially eligible subjects were excluded due to meeting exclusion criteria, and subsequently a total of 289 potentially eligible subjects were approached by the study team, of which 13 patients were enrolled and completed the study interview (Fig 1). Several reasons that potentially eligible subjects were not enrolled included declining to participate, being unable to reach for screening after initial approach, or not meeting full inclusion criteria during eligibility screening. The most commonly cited reason for not meeting full inclusion criteria was lack of an in-person visit to the Kaiser Permanente San Jose psychiatry clinic over the previous 12 months. We attributed this issue to the COVID-19 pandemic, which began during study recruitment, resulting in many patients having had only virtual visits with their provider over the preceding year. Those who declined to participate cited reasons of lacking time or lacking interest in participation.

### Procedure

The study team sent potentially eligible subjects a secure message that included a brief summary of the study, request for them to participate, and an option for them to opt out of further contact from the study team. To the recruitment email, we also attached an information sheet (S1 Appendix) that described the study in greater detail and provided key elements of informed consent. Eligible subjects that did not opt out were contacted by telephone by the study team approximately one week later. Interested subjects then completed an eligibility screen by phone, where they were also informed of department interests in seeking design feedback from patients after purchasing a new building. If they were eligible and still interested in participating, they were scheduled for a phone appointment where the terms of verbal informed consent were read to the subjects, and subjects were given the opportunity to ask questions or decline to participate. Subjects that provided verbal informed consent were enrolled, and then proceeded to a semi-structured interview by phone immediately afterward (S2 Appendix). Semi-structured interviews lasted 60–90 minutes, and were performed by two clinical psychiatrists (TL and HH). Enrollment was conducted on a rolling basis in parallel to data analysis (see below), and was halted when all members of the study team felt that saturation of themes had been reached. After completion of the interview, subjects were able to pick up a gift bag of hospital-branded items worth approximately $30 in total value.

Interviews were recorded using a digital voice recorder, and the files were stored in limited-access folders, on secure servers, behind the Kaiser Permanente firewall. Research data was stored separately from any identifiers, and any data transmitted electronically was encrypted during transmission. Participants were anonymized at enrollment. All interviews were transcribed verbatim, and transcripts were reviewed by a study team member for quality prior to analysis.

### Data analysis

Sensitizing concepts were utilized in the open, inductive analysis of transcript data; analysis was performed within a constructivist framework in order to be as inclusive as possible of each participants’ subjective experience of the topic. Rather than mapping gathered data onto pre-determined schemas, this framework allowed us to construct knowledge from the data and to generate themes apart from hypotheses researchers may have had. Two study team members had prior experience in qualitative research and use of this approach. Two study team members independently coded all transcripts initially; any specific locations, physical space elements, emotions, and patterns related to physical space and/or mental health recovery were highlighted as potential codes within the initial review of the transcript. Coding of each transcript occurred on a rolling basis, as interviews were being completed. The study team members iteratively reviewed codes with each other until consensus of codes for each participant was reached. Any patterns or associations noted across various participants’ codes were extracted as potential themes. Themes were reviewed by at least three study team members for investigator triangulation until saturation was reached. When codes from new interviews mapped to previously generated themes and did not appear to suggest novel themes, the study team determined that saturation was reached, resulting in the conclusion of study participant enrollment. Notes from each analysis discussion between team members were taken to provide an audit trail. Illustrative quotes were kept at all levels of analysis (initial coding, consensus coding, and theme extraction) for additional documentation. Reflexivity was maintained through open discourse in reflection meetings between study team members throughout the analysis as well as journaling by study interviewers after each interview was conducted. Our analysis followed SRQR (Standards for Reporting Qualitative Research) guidelines [12].

## Results

**Fig 2:**
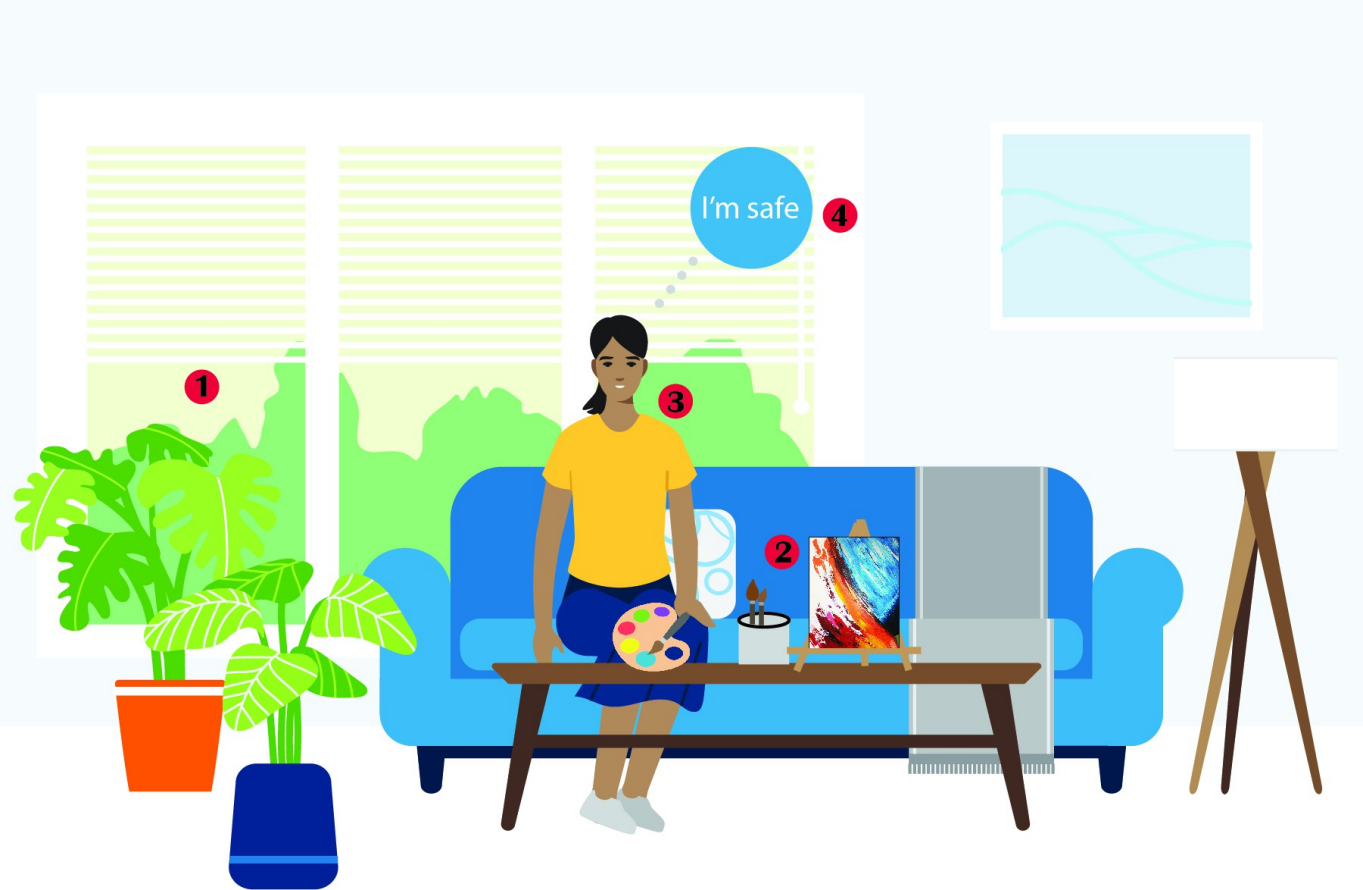
Key themes. A depiction of a hypothetical mental health recovery space that integrates key themes in this study, including 1) sensory design elements (for example, the presence of natural light and plants as a reminder of nature), 2) engagement intensity (engaging in a creative activity like painting), 3) social relational aspects (privacy and personal space), and 4) affective experiences (feeling of safety).

The sample was comprised of nine female and three male participants, and one unidentified-gender participant, between the ages of 26–64. Services that participants reported receiving at the clinic included medication management, individual psychotherapy, group psychotherapy, intensive outpatient programming (in the past, not concurrently with study participation), or combinations of these services. Self-reported race/ethnicities included Caucasian, Black, Asian, and Hispanic, with self-reported education levels from high school to post-baccalaureate certification. Self-reported household income also varied from US$ 10,000 to US$ 300,000 per year. We found four dimensions of physical environments that participants identified as significant to their mental well-being and recovery: 1) **sensory** design elements, 2) **engagement** intensity 3) social **relational** aspects and 4) the **affective experiences** evoked by being present in the space itself (summarized in Fig 2).

### Sensory elements

Sensory elements of physical space, including color, sound, and texture, were prominent in participants’ descriptions of the physical environment. Elements that most participants described as contributing to recovery included soft and neutral colors; natural light; natural sounds, like hearing the breeze; and other natural elements, such as plants. Seeing, hearing, and experiencing the sensory aspects of nature featured prominently in participants’ descriptions of environments that promoted recovery:

*HHWAV08*: *“I think my psychiatry is the beach. I love to go to the beach, but you cannot incorporate that in this treatment.”*

*TLWAV03: "I like a lot of light*. *Windows, a lot of natural [light] coming in. It’s very uplifting…"*

More than one-third of participants mentioned that human-made patterns and light could also contribute to recovery because these elements engaged participants’ attention, leading to a grounding effect. The calming influence of pattern and light was experienced by participants in different contexts, such as when looking at magazines, paintings, and shadows created by artificial light on roadsides.

*HHWAV06*: *"I enjoy looking at the paintings, things—if I can find anything different within them each time I see them…calm, distracted from the negative."*

*HHWAV10*: *"…when you’re driving, you see different lights, you see different—you feel different textures while hitting different textures on the road that keeps you distracted…like grounded."*

In contrast, sensory experiences that were described across most interviews as having an adverse effect on mental health and recovery included those that were dissonant, jarring, or unnatural. Examples of deleterious sensory stimuli included clashing colors and patterns, harsh lighting, clutter and disorganization, and loud noises such as outside traffic or television and radio.

*TLWAV05: "If it has a lot of patterns*, *it gives me anxiety. It’s too busy for me. And then, solid is more soothing for me. Kind of it can, like–it’s [exhales] "I can breathe."*

*TLWAV04: "I feel like that makes me feel stressed when–like, being in closets or anything that’s cluttered and small."*

*TLWAV03:"…elements that are more industrial, that are cold. Metal or metallic presents a feel that is more sterile and…Less engaging and feeling comfortable. You know, black and white, contrasting, sharp contrasts. And more sharp edges. I think that kind of design is not so good."*

### Engagement intensity

Places where participants engaged in a process of creation (e.g., crafting, painting, sculpting) or physical exercise were associated with recovery. Art studios, gyms, workshops, dance studios, or outdoors were described as places that promoted mental health because the interactive, artistic, or athletic engagements that occurred in these spaces provided a sense of accomplishment, progress over time, or distraction that helped with recovery. Some participants attributed value in the ability to personalize these spaces to their own preferences.

*HHWAV03: "I feel creative when I’m in here because I’m around all the fabrics and the different things that I use. It helps me when I’m making something…you kind of get out of your head a little bit and use different parts of your brain, the creative side. And then you get to take the pictures or whatever you did home or back to your room and hang them up…it carries it into your living space*."

*TLWAV04: "…it was a distraction also. So, it was kind of like perfect being outside and doing something I learned to love, which was gardening*."

*TLWAV01: “I have a work space that I just recently built that feels pretty comfortable when I’m there. Tools, like saws and hammer and screw driver and all that kind of stuff… I built this space myself. I think that has some value to it. And it’s just my space that I can go to do things*.”

Places that engaged participants in less active ways requiring participants to interact with the environment just enough to complete specific tasks—such as commuting or grocery shopping—emerged as important sites for recovery. Interestingly, these participants specifically associated these less-engaging places with breakthroughs in mental health recovery (i.e., uncovering a new insight), rather than simply promoting mental well-being.

*HHWAV04*: *"I am delighted if I’m able to look at or understand something differently than I had before…I’m somewhere, but I’m not really interacting with anybody directly. Out in driving, I’m watching the traffic, but I guess I’m interacting with traffic, but I’m not socially interacting with traffic. Walking. If it’s a trail, I’m interacting with what’s around me, not really people… It’s boredom, but not being distracted."*

This suggests that environments promoting a state of “intermediate” engagement–significant enough to impede ruminations but not significant enough to interfere with constructive thought patterns–might facilitate progress in mental health recovery.

### Social relational elements

Participants’ proximity to other humans in a space was a third element that contributed to perceptions of mental health in an environment. Participants overwhelmingly preferred spaces that allowed them to maintain their personal space. Being in crowded areas or being too close to others evoked negative emotions such as anxiety or feeling exposed.

*HHWAV10: "The mall…once it gets really busy and picks up, I have to leave…everybody would just be walking in everyone’s personal space*. *It’s always crowded"*

*TLWAV03: "It was definitely an experience physically sitting too close to the therapist, to the doctor, and didn’t like it. And I felt too boxed up and too tight and too unable to express myself…physically I just had to suck it up and be quiet. And I couldn’t let my feelings out. And the space, the proximity was not good*. *It was awkward and uncomfortable."*

Several participants also highlighted privacy as an important feature, although how privacy was achieved varied across different spaces:

*TLWAV03: “cubicles, those are just too open and lack privacy and more squished together.”*

*TLWAV04: “I like actually being outside…the privacy, the quiet, kind of like the distance from everything*.”

The quality of the interactions with other humans was an important element of space that promoted mental health. When participants described spaces that were supportive of recovery, the humans in those spaces were people who listened and cared. For example, one participant described their psychiatrist as their safe space:

*TLWAV05*: *"One of my favorite spaces is Dr. M’s office…Dr. M in general. He’s very, very soothing for me. He’s very helpful. He’s my safe spot or safe place…"*

In contrast, another participant discussed how their childhood home did not support their recovery because the humans inhabiting that space did not actively listen or want to hear what the participant was feeling:

*HHWAV10*: *"Moments at my parents’ house…they never want to hear anything I say, when it comes to how I feel. I mean, they’re happy I’m there, but they don’t want to hear about anything negative that happened, say about how I feel"*

The quality of the interactions with other people within a space, as well as the ability to maintain privacy and personal space, influenced the extent to which a space could promote recovery.

### Affective experiences

Most participants described physical spaces evoking emotions or emotional experiences, which could in turn influence mental health. For example, one participant noted a special connection to the ocean, which included not just a feeling of calm, but also an emotional sense of timelessness which helped the participant to gain a better outlook on their mental health recovery:

*TLWAV02: “… something that helped me a lot is to go to a place that was so much bigger than myself. It was grander than I am. With the ocean, it’s just–it’s huge…And to kind of put my issues in perspective to me…it was reliable.*”

In another example, a participant associated being in bed with an emotional experience of feeling trapped, which perpetuated negative mood.

*TLWAV02: "…it can be both sometimes. And, you know when you’re low and when you’re down, it’s really easy to stay there because you don’t have to do anything but be there. And it’s shitty, but I don’t have to do anything about it. And being in my bed, it’s warm, and it’s cozy, and I don’t have to do anything except for feel terrible. And it’s my bed, and I do love it…I just feel like it’s a trap*."

One participant described feeling safe in the provider’s office because the privacy and neutrality of the space focused attention on the therapeutic process:

*HHWAV10: "it’s private, it’s one-on-one with you and the therapist or psychiatrist or psychologist, I feel like it’s a safe haven…I like how the rooms are neutral, how they don’t draw too much attention, how I’m able to focus on my conversation*."

Other environments evoked feelings of anxiety, depression, lack of control, claustrophobia, or even triggered reminders of past trauma.

*HHWAV11: "It just reinforced the symptoms of depression…My room is a mess*."

Interestingly, there was one participant who noted that he/she/they had not considered the connection between physical space and mental health prior to the interview:

*HHWAV12: "I don’t think location, a difficult location is that important. However, that’s probably because I have a pretty good physical location to live in…I don’t use them per se, I exist in them…I guess I’ve never really thought about how those spaces do affect me, but perhaps they do*”

Affective responses to a location seemed to offer a valence, therefore, to the perception of how helpful a place was to a participant’s mental health recovery.

## Discussion

This study identified several key themes regarding the physical environment that participants found to be impactful in mental health recovery–sensory design elements, engagement intensity, social relational aspects, and affective experiences. The sensory design elements, social relational aspects, and affective experiences evoked had both positive and negative valence; on the other hand, engagement intensity was generally considered to be positive. We note that our findings are consistent with other investigations in the area of human-environment interfaces. A recent systematic review identified 16 distinct mechanisms across CES domains, four of which mirrored our findings closely: (1) formative mechanisms, defined as the change of individuals’ perceptions that is relatively instantaneous over short periods of time (mapping to our sensory element); (2) creative mechanisms, defined as the experience of situations that inspire creativity, work, and freedom (engagement element); (3) cohesive mechanisms, defined as the development of meaningful relationships between people (social element); and (4) evolutive mechanisms, defined as the gradual change of individuals’ personality, mood, and feelings over time (affective element) [6]. It is conceivable that the journey of mental health recovery might engage a specialized subset of these known human-environmental interaction mechanisms.

All participants commented on sensory elements of the physical space, including colors, contrast, clutter, textures, lighting, and symmetry. Perhaps these elements were the most noticeable components of what comprises a physical space. In greenspace literature, Gianfredi et al. [7] noted that an increased number of urban greenspaces were found to be predictors of decreased stress. This is consistent with the findings of this study, in which participants described several outdoor locations (parks, beaches, trails), as well as nature-related elements found indoors including windows allowing sunlight and the presence of plants to exert an overall positive influence on mental health recovery.

Over half of the participants discussed engagement intensity within the physical space. Several participants referenced exercise, dancing, or other types of movement-based activities, which again correlates with greenspace literature in which opportunities for physical activities created by greenspace are associated with improved mental health [8]. Other participants mentioned activities such as crafting, painting, sculpting, or working with tools, suggesting that physical spaces conducive to the process of creating are beneficial in mental health recovery. The positive associations between activities such as exercise and art to mental health have been widely studied; existing research linked exercise to positive mental health outcomes [13], and mindfulness-based art therapy [14] is a commonly used intervention. Lastly, we also noted mention of less-engaging places like commuting cars or grocery stores that some participants felt contributed to moments where they experienced the most progress in their mental health recovery. This seems consistent with social research suggesting a potential link between boredom and creativity, which in turn may allow for the generation of new mental health insights for recovery [15].

Another key theme was the social relational elements of the physical space. A positive finding within greenspace literature is that greenspace appeared to decrease the perception of loneliness and increase perceptions of security and social belonging [2]. In contrast, participants in this study tended to value personal space, which was associated with privacy and confidentiality. We also found that quality of the social connections found within a specific environment seemed to matter more than absolute social exposure, a finding also consistent with previous literature [16].

Participants’ discussions of affective experiences highlight that there was a segment of the population where this was a more prominent part of their environmental experience than for others. While there was overlap between some sensory and affective themes, such as natural elements providing soothing sensory cues and a feeling of relaxation, we note this was not always the case. In one example provided above, the participant described the sensory experience of being in bed at home as comfortable and warm, but the affective experience as depressive and feeling stuck “in a trap.” This suggests that the sensory and affective elements of an environment can be independent of each other. Indeed, the CES literature also draws a distinction between sensory (formative) and affective (evolutive) mechanisms of human-environment interaction [6].

This is the first qualitative study focused on patient perspectives of physical space in outpatient mental health recovery. Limitations to our findings include the qualitative nature of the study which may overlook other patient perspectives. Our participant population was skewed toward female gender, for example, as often occurs in clinic settings where depression and anxiety disorders predominantly affect females over males (~2:1 ratio) [17]. We also did not enroll non-English speaking participants, as our clinical interviewers spoke only English, and consequently may have missed other important cross-cultural perspectives. The context of our design orientation (participants and study team being aware of the sponsoring institution’s investment in a new clinic building) may also have limited our study; however, we feel this real-world constraint also contributed toward generating actionable findings. Last, study interviews occurred during the COVID-19 pandemic (2020–2021) where participants may have been more limited in access to physical spaces around them, which may have affected their responses.

Traditional mental health treatment occurs within the confines of the clinic environment. However, even within that frame, much of the recovery process in outpatient care still occurs between clinic visits, in environments external to the physical space of the clinic. Although our findings inform design elements that can be useful building blocks for HCD-based approaches to the construction of healing spaces, we note that the four dimensions of environmental impact can also support patients and clinicians seeking to harness potential benefits of physical environments encountered in daily living–in other words, a design-oriented approach to a patient’s mental health journey. For example, a clinician and patient may augment psychotherapeutic interventions with a brief overview of a patient’s daily physical environment “regimen,” and use the above dimensions as a checklist to identify ways a patient could enhance their current environmental interactions. As the COVID-19 pandemic has only shifted treatment further outside of traditional clinic settings with the rise of telehealth and virtual offerings for mental healthcare, our findings may guide a new path for mental health providers and patients to collaborate on optimizing mental health recovery outside of the clinic.

## Supporting information

S1 Checklist (DOCX)

S1 Appendix Information sheet in recruitment email. (PDF)

S2 Appendix Interview guide. (DOCX)
