# Supplementary material for: The impact of physical environments on outpatient mental health recovery: A design-oriented qualitative study of patient perspectives
Source: PLoS One. 2023 Apr 19;18(4):e0283962. doi: 10.1371/journal.pone.0283962 (PMC10115290; doi:10.1371/journal.pone.0283962)
Supplement: S1 Appendix — (PDF) [file pone.0283962.s002.pdf]

# *Perspectives on Physical Space in Outpatient Mental Health*

## Frequently Asked Questions:

### Are you eligible?

- Adult 18-85 yrs old.
- Receiving Mental Health Treatment at KP San Jose.
- Able to read, speak, and write conversational English.

### What will happen if I take part in this study?

You will participate in one 15 minute interview to determine eligiblity and if eligible, one 90-minute interview with the study team by phone.

### Do I have to participate in the study?

Participation is completely voluntary. You are free to refuse, and your decision of whether to participate will not affect your medical care. If you decide to participate, you are free to change your mind and discontinue participation at any time.

### What are the potential risks and discomforts?

Risks associated with this study are minimal. The only discomfort you may experience is feeling inconvenienced by having to complete the interview; however, our research staff will make every reasonable effort to accomodate your schedule.

### How will my privacy be protected?

Privacy is very important to us at Kaiser Permanente. Your name and any other information that allows you to be identified will not be linked to research data. Investigators will ensure that the link between your name and these study numbers will never be released outside the hospital/study site unless required by law.

### Are there any benefits?

There are no direct benefits but we hope that the results of this study may benefit you and other patients in the future.

### Will I be paid to take part in this study?

You will be compensated with a $30 gift card for participation.

If you are interested, please contact Dr. Honor Hsin at **408-362-3750.**
