# Supplementary material for: The impact of physical environments on outpatient mental health recovery: A design-oriented qualitative study of patient perspectives
Source: PLoS One. 2023 Apr 19;18(4):e0283962. doi: 10.1371/journal.pone.0283962 (PMC10115290; doi:10.1371/journal.pone.0283962)
Supplement: S2 Appendix — (DOCX) [file pone.0283962.s003.docx]

**Interview Guide**

Thank you for participating in this study interview. Please take your time to respond to the questions. As a reminder, you may ask to pause the interview, skip questions, or stop the interview completely at any time. Let’s begin.

[**Introduction**] Recovery from mental health conditions is a personal journey. Often, this journey involves progression through various physical spaces, such as the clinic office during outpatient visits, or a routine commute where coping skills may be practiced. We would like to learn more about how people like you think about physical space and design elements as part of your mental health recovery and outpatient care.

[**Question 1**] Can you think of one example of a physical space that was **very good** for your mental health? This could be indoors or outdoors, homes (such as a house, apartment, or specific rooms), communal areas (such as museums, restaurants, gyms or specific facilities), open spaces (such as park benches or trails), or any other place that comes to mind. Describe it to me.

[**Record here**]

1a. In architecture, there are several design elements that characterize a space. These include color choice, contrast between colors, degree of emptiness vs clutter, texture or patterns, lighting, and symmetry, among other elements. What elements of this space make it good? How do those elements make you feel?

[**Record here**]

1b. Can you think of one example of a physical space that was **very bad** for your mental health? This could be any place that comes to mind. Describe it to me.

[**Record here**]

1c. In architecture, there are several design elements that characterize a space. These include color choice, contrast between colors, degree of emptiness vs clutter, texture or patterns, lighting, and symmetry, among other elements. What elements of this space make it bad? How do those elements make you feel?

[**Record here**]

[**Question 2**] Let’s think about your own journey of mental health recovery, the process by which you are working on improving your mental health. From when you first embarked on your journey of recovery to now, what physical spaces play an important role in **improving or enhancing** your recovery? These could be spaces that you pass through during a typical day, or maybe only on special days. This could be your care provider’s office, a room at work, a room at home, a different clinic office, a certain park, the gym, your car, a social gathering place, etc. Please name at least 3.

[**Record here**]

2a. How did you use the physical space of each place you identified? How did these spaces make you feel?

[**Record here**]

[**Question 3**] From when you first embarked on your journey of recovery to now, what physical spaces play an important role at **worsening or delaying** your recovery? These could be spaces that you pass through during a typical day, or maybe only on special days. This could be your care provider’s office, a room at work, a room at home, a different clinic office, a certain park, the gym, your car, a social gathering place, etc. Please name at least 3.

[**Record here**]

3a. How did you use the physical space of each place you identified? How did these spaces make you feel?

[**Record here**]

[**Question 4**] Think back to specific times when you progressed **the most** in your recovery (gained a new insight, practiced a new skill, learned something new, or made a different choice for your health). Where were you during the moments you made the most progress in your recovery? How did you use this space? How did it make you feel?

[**Record here**]

4a. Where were you during the moments you found it **hardest** to make progress in your recovery (gain a new insight, practice a new skill, learn something new, or make a different choice for your health)? How did you interact with this space? How did it make you feel?

[**Record here**]

[**Question 5**] Thinking about our Kaiser Permanente San Jose outpatient mental health clinic, how important is this place to your mental health recovery?

[**Choices: very important, somewhat important, somewhat unimportant, irrelevant]**

5e. Thinking of your physical space experience at the clinic, can you share what you are usually thinking or feeling at each of the stages below?

5e1. Entryway as you walk into the building

[**Record here**]

5e2. Reception as you check in for your appointment

[**Record here**]

5e3. Waiting seats as you wait for your appointment to begin

[**Record here**]

5e4. Clinic corridor as you walk to the office for your appointment

[**Record here**]

5e5. Clinic office or group room where you receive treatment

[**Record here**]

5e6. Exit route as you leave the office, corridor, and building

[**Record here**]

5e7. How could your experience at each stage be improved?

[**Record here**]

Last, we would like to learn some basic information about you. This part should only take a few minutes of our remaining interview time. You may skip any questions you wish.

What is your age?

What is your identified gender?

What is your identified race and/or ethnicity?

What is your highest level of education?

What is your approximate annual household income?

Which of the following psychiatry department services have you ever used at Kaiser Permanente San Jose (please choose as many as applicable):

- Outpatient medication management?
- Outpatient individual psychotherapy?
- Outpatient group therapy?
- Intensive outpatient program?
